# Editorial: Exploring the therapeutic effects of synthetic, semi-synthetic and naturally derived compounds against cancer

**DOI:** 10.3389/fphar.2023.1251835

**Published:** 2023-08-22

**Authors:** Liang Feng, Junfei Gu, Yanjun Yang, Bing Yang, Ruyu Shi

**Affiliations:** ^1^ State Key Laboratory of Natural Medicines, Department of Traditional Chinese Pharmacy, China Pharmaceutical University, Nanjing, China; ^2^ School of Chinese Medicine, School of Integrated Chinese and Western Medicine, Nanjing University of Chinese Medicine, Nanjing, Jiangsu, China

**Keywords:** chemopreventive agent, natural medicine, anticancer, potential target discovery, action mechanisms

There are serious challenges in the treatment of cancer, which can sometimes be life-threatening. Indeed, natural compounds or naturally-derived compounds have been recommended in treating cancer because of their relative safety and low cost (Messeha et al., 2022). In recognition of this important and growing field of research, we hereby present a Research Topic of primary research articles and reviews on the Research Topic of Focus on compounds or compositions with potential activities including synthetic, semi-synthetic, structurally modified, natural sources, and Chinese medicines to explore their beneficial effects on cancers and explore its potential mechanisms of action.

Natural compounds are being recognized as promising candidates for anticancer therapy due to their safety and multitarget intrinsic features. In addition, numerous studies have found that a natural compound may have multiple active targets rather than only one unique target (Chen et al., 2021; Fakhri et al., 2021). Therefore, many natural products may play multiple roles in cancer treatment. Plant extracts, in particular, have been for centuries used as therapeutic agents (Lee et al., 2021), and are still object of extensive research for their manifold properties and mechanisms of action, that make them promising molecules for novel cancer treatment strategies.

This Research Topic brings together works that make significant contributions to the advancement of research in the field of cancer therapy, taking into account the challenges involved in establishing effective treatments and the crucial role synthetic, semi-synthetic and naturally derived compounds plays in the development and progression of cancer.

Zengshengping (ZSP) is a widely used antitumor traditional Chinese medicine compound in clinics. It is a Chinese herbal medicine formulation. Sun et al. used the Lewis lung cancer model, H&E staining, ELISA, immunohistochemistry and other techniques to investigate the development of lung cancer, tumor cell proliferation, immune organ index, inflammatory factor level to evaluate the efficacy of ZSP in the prevention and treatment of lung cancer. Protein immunoblotting, immunohistochemistry and other methods were used to investigate the physical barrier and immune barrier of the intestinal mucosa, 16Sr high-throughput sequencing technology was used to investigate the intestinal mucosal biological barrier of Lewis lung cancer model mice and urethane-induced lung cancer model mice, and evaluate the effect of ZSP on intestinal mucosa of lung cancer mice. “Lung cancer”, “gut-lung axis”, and “treating the lung from the intestine” are three aspects of progressive development, and the role of ZSP in the prevention and treatment of lung cancer is expounded.

Chen et al. showed that the expression of PUMA (p53 upregulated modulator of apoptosis) was upregulated upon platycodin D treatment. Knockdown of PUMA resulted in attenuation of platycodin D-induced apoptosis, indicating that PUMA upregulation is essential for platycodin D to induce apoptosis. The induction of PUMA expression by platycodin D treatment was through activation of AP-1 since mutation of AP-1 binding site in the PUMA promoter abolished the PUMA promoter activity. Moreover, knockdown of JNK1, but not JNK2, significantly abolished the phosphorylation of c-Jun at Ser63 (a component of AP-1), decreased the platycodin D-induced expression of PUMA and cleaved caspase 3, indicating that platycodin D inhibits JNK1/AP-1 signaling pathway for apoptosis induction in suppression of NSCLC (non-small cell lung cancer) growth, providing a new mechanism of how platycodin D suppresses NSCLC.

Li et al. provided existing evidence and molecular mechanism of Tanshinone IIA’s anti-HCC effect but also reviews the liverprotective effect of TsIIA and its impact on liver fibrosis, NAFLD, and other risk factors for liver cancer. In addition, we also conducted network pharmacological analysis on TsIIA and HCC to further screen and explore the possible targets of TsIIA against hepatocellular carcinoma. It is expected to provide a theoretical basis for the development of anti-HCC-related drugs based on TsIIA.

Rita Volpe et al. studied the effect of hydroalcoholic extract from dried leaves of *Buxus sempervirens* (BSHE) on four human cell lines (BMel melanoma cells, HCT116 colorectal carcinoma cells, PC3 prostate cancer cells, and HS27 skin fibroblasts) to ascertain its possible antineoplastic activity. BSHE appeared to promote autophagic flow with its following blockade and consequent accumulation of autophagosome or autolysosomes. The antiproliferative effects of BSHE also involved cell cycle regulators such as p21 (HS27, BMel, and HCT116 cells) and cyclin B1 (HCT116, BMel, and PC3 cells) whereas, among apoptosis markers, BSHE only decreased (30%–40% at 48 h) the expression of the antiapoptotic protein survivin. It was concluded that BSHE impairs autophagic flow with arrest of proliferation and death in both fibroblasts and cancer cells, being the latter much more sensitive to these effects.

Xu et al. screened total of 90 genes for Dendrobin A anti-PDAC, and a PPI network for Dendrobin A anti-PDAC targets was constructed. Notably, a scale free module with 19 genes in the PPI indicated that the PPI is highly credible. Among these 19 genes, PLAU was positively correlated with the cachexia status while negatively correlated with the overall survival of PDAC patients. Through molecular docking, Dendrobin A was found to bind to PLAU, and the Dendrobin A treatment led to an attenuated PLAU expression in PDAC cells. Based on clinical tissue arrays, PLAU protein was highly expressed in PDAC cells compared to normal controls, and PLAU protein levels were associated with the differentiation and lymph node metastatic status of PDAC. *In vitro* experiments further showed that Dendrobin A treatment significantly inhibited the proliferation, migration, and invasion, inducing apoptosis and arresting the cell cycle of PDAC cells at the G2/M phase.

In the study by Chen et al. Cinobufacini injection (CI), an aqueous extract of *Cutis Bufonis*, is clinically used for cancer therapy in China, but its molecular mechanism for the treatment of osteosarcoma (OS) remains unclear. We constructed U2OS ectopic subcutaneous tumor model to verify the anti-OS effect of CI *in vivo*. Meanwhile, cell proliferation of U2OS and MG63 cells was monitored *in vitro* using the CCK-8 assay, colony formation and morphological changes. Cell cycle arrest and apoptosis were detected by flow cytometry and Western blot, which showed that CI significantly inhibited proliferation, induced cell cycle arrest and apoptosis in human OS cells. The further RNA-seq results identified that the Hippo signaling pathway was involved in the anti-OS effect of CI. YAP/TAZ are two major components of the Hippo pathway in breast cancer and are positively regulated by prolyl isomerase PIN1, we assessed their role in OS using both clinicopathological sections and western blots. CI also inhibited PIN1 enzyme activity in a dose-dependent manner, which resulted in impaired PIN1, YAP, and TAZ expression *in vitro* and *in vivo*. Additionally, 15 potential compounds of CI were found to occupy the PIN1 kinase domain and inhibit its activity. In summary, CI plays an anti-OS role by down-regulating the PIN1-YAP/TAZ pathway.

Min et al. found that the atorvastatin use could increase the risk of colorectal cancer (odd ratio (OR) = 1.041, *p* = 0.035 by fixed-effects inverse variance weighted (IVW) method (IVWFE), OR = 1.086, *p* = 0.005 by weighted median; OR = 1.101, *p* = 0.048 by weighted mode, respectively). According to the weighted median and weighted mode, atorvastatin could modestly decrease the risk of liver cell cancer (OR = 0.989, *p* = 0.049, and OR = 0.984, *p* = 0.004, respectively) and head and neck cancer (OR = 0.972, *p* = 0.020). Besides, rosuvastatin use could reduce the bile duct cancer risk by 5.2% via IVWEF method (OR = 0.948, *p* = 0.031). No significant causality was determined in simvastatin use and pan-cancers via the IVWFE or multiplicative random-effects IVW (IVWMRE) method if applicable (*p* > 0.05). There was no horizontal pleiotropy observed in the MR analysis and the leave-one-out analysis proved the stability of the results.

Zhang et al. identified EGFR from more than a dozen predicted targets as a protein that directly binds to acacetin. Moreover, acacetin affected the level of phosphorylated EGFR. *In vitro*, acacetin promoted the apoptosis of GC cells. Importantly, EGFR agonists reversed the inhibitory effects of acacetin on the STAT3 and ERK pathways. *In vivo*, acacetin decreased the protein levels of pEGFR in tumors, resulting in increased GC xenograft tumor regression without obvious toxicity.

In a case report study by Shen et al. they described a rare case of idiopathic pulmonary fibrosis associated with the use of nab-paclitaxel and carboplatin in ovarian cancer. During treatment, it is necessary to maintain a high level of vigilance for patients with interstitial pneumonia and engage the attention of clinicians to improve medication safety. Early diagnosis and anti-fibrosis therapy can reverse lung damage.

During the last few decades, an overwhelming number of both preclinical and clinical studies have demonstrated the potential of synthetic, semi-synthetic and naturally derived compounds in the prevention and treatment of cancer (Scott et al., 2013; Ren and Kinghorn, 2019). Emerging evidence suggests that natural bioactive compounds confer anticancer activities via impacting carcinogen metabolism, oxidative stress, inflammation, cell proliferation, apoptosis, autophagy, cell cycle regulation, epigenetic alteration, invasion, migration, adhesion, angiogenesis, and metastasis by regulating a plethora of cell signaling molecules, mediators and modulators (Rios et al., 2009; Von and vollmar 2013; Deng et al., 2019; Xiao et al., 2022).

In order to achieve the desired level of protection, it is imperative that researchers take into account all relevant mechanisms and explore a range of comprehensive measures or drug combinations. The development of molecular biology technology has led to the research of targeted therapy using natural products or derivatives that are highly selective for tumor as carriers, it is necessary to study the optimal dose for protecting against different tumors, and chemically coupling these factors into biological treatments (Tobeiha et al., 2021). In addition, direct delivery of natural product to the tumor site can not only reduce the amount of natural product needed but also improve the efficacy and reduce adverse reactions. This opens up new ideas for the study of protective measures against cancer. On the one hand, there are still existing disparities between remarkably anticarcinogenic effects in preclinical models and negative results in real-world observational studies. On the other hand, the potential confounding factors that existed in observational studies could also overlap the real influence of statin use in cancers (Zhi et al., 2020). Therefore, future well-designed double-blind randomized controlled trials are warranted to validate the existing correlations we determined.

Overall, the nine papers published on this Research Topic highlighted several natural products or traditional Chinese medicine that can be characterized appropriately to enhance immunotherapy of diverse human cancers. In summary, natural products with well-characterized structures or traditional Chinese medicines with well-defined functions have great potential in the treatment of cancer. They can be used in combination with other immunotherapies as chemoprevention, chemotherapy or adjuvant therapy to manage various cancers. However, in-depth studies with more physiologically and pathologically relevant models are needed to determine its efficacy and clinical effectiveness. Therefore, further verification of the efficacy of these natural bioactive substances and traditional Chinese medicines will provide more convincing evidence for future cancer treatment or adjuvant therapy.
